# Genetic Diversity and Phylogeography of a Turf-Forming Cosmopolitan Marine Alga, *Gelidium crinale* (Gelidiales, Rhodo-Phyta)

**DOI:** 10.3390/ijms24065263

**Published:** 2023-03-09

**Authors:** Ga Hun Boo, Antonella Bottalico, Line Le Gall, Hwan Su Yoon

**Affiliations:** 1 Department of Biological Sciences, Sungkyunkwan University, Suwon 16419, Republic of Korea; 2 Department of Biosciences, Biotechnologies and Environment, University of Bari “A. Moro”, Via E. Orabona 4, 70125 Bari, Italy; 3 Institut de Systématique, Evolution, Biodiversité (ISYEB), Muséum National d’Histoire Naturelle, CNRS, Sorbonne Université, EPHE, 75005 Paris, France

**Keywords:** biogeography, cosmopolitan distribution, *Gelidium crinale*, geographical structure, Pleistocene relict, red algae

## Abstract

Cosmopolitan species are rare in red algae, which have a low-dispersal capacity unless they are dispersed by human-mediated introductions. *Gelidium crinale*, a turf-forming red alga, has a widespread distribution in tropical and temperate waters. To decipher the genetic diversity and phylogeography of *G. crinale*, we analyzed mitochondrial COI-5P and plastid *rbc*L sequences from collections in the Atlantic, Indian, and Pacific Oceans. Phylogenies of both markers statistically supported the monophyly of *G. crinale*, with a close relationship to *G. americanum* and *G. calidum* from the Western Atlantic. Based on the molecular analysis from these materials, *Pterocladia heteroplatos* from India is here merged with *G. crinale*. Phylogeny and TCS networks of COI-5P haplotypes revealed a geographic structure of five groups: (i) Atlantic-Mediterranean, (ii) Ionian, (iii) Asian, (iv) Adriatic-Ionian, and (v) Australasia-India-Tanzania-Easter Island. The most common ancestor of *G. crinale* likely diverged during the Pleistocene. The Bayesian Skyline Plots suggested the pre-LGM population expansion. Based on geographical structure, lineage-specific private haplotypes, the absence of shared haplotypes between lineages, and AMOVA, we propose that the cosmopolitan distribution of *G. crinale* has been shaped by Pleistocene relicts. The survival of the turf species under environmental stresses is briefly discussed.

## 1. Introduction

“Long-range dispersal of seaweeds does exist, but it is an exception rather than the rule. If it were the rule, the world’s seaweed floras would show similar latitudinal gradients in species composition in the oceans and on both hemispheres. This is, however, not the case.”—van den Hoek [1].

Coastal ecosystems face manifold problems stemming from climate change, and large canopy-forming species have gradually been replaced by small turf-forming species or introduced aliens [2,3]. Although turf-forming species often contribute less to the structure and function of coastal ecosystems, in the case of the absence of canopy species, they may take over an important function as habitat formers [4,5]. Our knowledge has yet to figure out the genetic diversity and geographical distribution of turf-forming species with a global distribution.

*Gelidium crinale* (Hare *ex* Turner) Gaillon (Gelidiales) is a turf-forming, small-sized (less than 7 cm tall) red alga. Plants are prostrate to erect, are terete at the base and compressed upwards, alternately or palmately branched, and have terasporangia and cystocarps borne apically [6,7,8]. They are perennial and occur year-round [9,10]. However, erected plants predominate during the warm season and are usually buried beneath sand during the cold season [10]. Asexual reproduction by tetraspores is common, but sexual gametophytes only occurred in 6% of total collections from the Atlantic Iberian Peninsula [11]. Plants commonly propagate from prostrate axes and regenerate from truncated axes and branches [10,12]. *Gelidium crinale* inhabits the intertidal sandy, rocky shore to the shallow subtidal zone [6,10]. It forms almost monospecific turfs covering rocky surfaces of around 1 m^2^ in Spain [11]. Despite having a lower dispersal capacity than other red algae, *G. crinale* widely occurs in the Atlantic, Indian, and Pacific Oceans [8,13,14].

*Gelidium crinale* has a wide morphological variation in thallus size and branching patterns [10,15,16]. Several infraspecific varieties have been reported, but they are no longer supported by recent molecular studies [8,17]. However, based on *cox*1 and *rbc*L phylogenies, *G. longipes* J.Agardh, an allegedly endemic species to New Zealand, was revised as *G. crinale* subsp. *longipes* (J.Agardh) W.A. Nelson and G.H. Boo [18]. Since its first description, *Pterocladia heteroplatos* (Børgesen) Umamaheswara Rao and Kaliaperumal from India has also been investigated as being the same species as *G. crinale* [19,20]. It was originally known as *Gelidium heteroplatos* Børgesen from specimens in Malabar Hill, Mumbai, on the western coast of India [19]. Later, based on unilocular cystocarps from a specimen likely collected on the eastern coast of India, it was transferred to *Pterocladia* [21]. It commonly occurs on the middle eastern coast of India and is harvested for the agar industry [22]. *Pterocladia heteroplatos* has also been recorded in Australia, East Africa, the Red Sea, and the Southeast Asian regions [14]. However, there are not yet any DNA sequences available from the material of the Indian species.

Molecular markers for examining the biogeography of widespread species are variable enough to display the genetic diversity of wide-ranging populations and also have a species-level resolution to circumscribe the boundary of the species. The ribulose-bisphosphate carboxylase large subunit (*rbc*L) in plastid and cytochrome c oxidase subunit I (*cox*1) in mitochondria has been widely used for the identification of species and the examination of genetic variation within red algal species [23,24]. Of five markers used in the phylogeny of the Gelidiales, both *rbc*L and *cox*1 represent the highly informative values at the species level [25]. However, COI-5P, a short region of the *cox*1 (around 664 bp), is similarly variable to *cox*1 and turns out to be the most suitable marker at the population level, given the number of haplotype and nucleotide diversity among geographical populations of *Gelidiella fanii* S.-M.Lin, the Indo-Pacific species in the Gelidiales [26]. The COI-5P also has a higher mutation rate than *rbc*L, which is useful for uncovering phylogeographic structure and genetic diversity [8,13,18]. Conversely, nuclear genes, e.g., the internal transcribed spacer 2 (ITS2), revealed lower resolution of the network than *cox*1 in two *Gelidiophycus* species [27] and have rarely been used in the biogeographic studies of red algae.

Both *cox*1 and *rbc*L sequences of *G. crinale* specimens from the type locality (Ilfracombe, Devon, United Kingdom) and a nearby location (Sidmouth, Devon) provided a baseline for the identification of the species [8,13]. Subsequent molecular studies demonstrated its occurrences in the Mediterranean, eastern Atlantic [8,11], and western Atlantic [13,28]. The occurrence of *G. crinale* in Asian and Australasian regions is also confirmed molecularly [7,8,13,29]. A haplotype network of *cox*1 has revealed some geographical structures associated with Asia, Australia, and Europe-America [8]. However, the genetic diversity and structure of *G. crinale* have yet to be examined at a global scale.

*Gelidium crinale* is interestingly a unique species with a cosmopolitan distribution among about 146 species of *Gelidium* [14]. The aims of the present study were to examine the genetic diversity and population structure of *G. crinale* and to decipher phylogeographic patterns to infer the cause of its cosmopolitan distribution. We analyzed sequences of both COI-5P and *rbc*L from wide-ranging collections of *G. crinale* and investigated population structures using COI-5P sequences. We discussed the ecological strategy of *G. crinale* under various environmental stresses for a better understanding of survival in changing situations of coastal ecosystems.

## 2. Results

### 2.1. Phylogenies and Divergence Time

**Figure 1:**
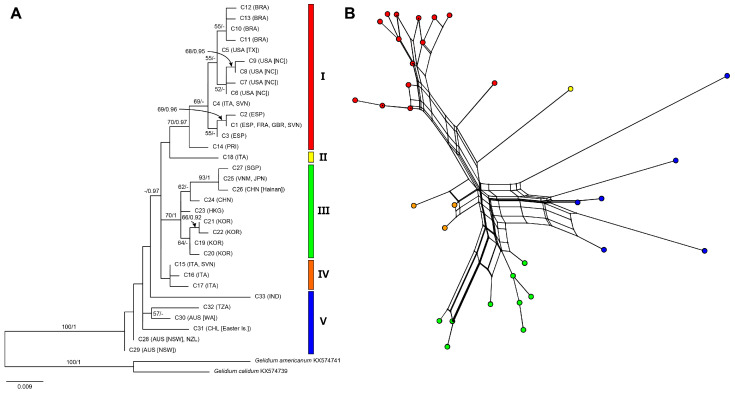
Maximum likelihood (ML) phylogeny (**A**) and split network (**B**) of *Gelidium crinale* using 33 unique mitochondrial COI-5P sequences. ML bootstrap values (≥50%) and Bayesian posterior probabilities (≥0.9) are shown at branches. Dash indicates values <50 or <0.9.

The final dataset comprised 167 sequences including 104 previously published sequences (62 COI-5P and 42 *rbc*L): 118 COI-5P (495 bp) and 49 *rbc*L (1266 bp), including two outgroups. Because multiple identical sequences do not provide any information and also slow the analyses, we only used unique sequences for phylogenetic analyses (35 COI-5P and 27 *rbc*L). Due to the low quality and degradation of DNA from the holotype specimen of *Pterocladia heteroplatos* (*Børgesen 5275*), only a partial *rbc*L sequence (124 bp) was obtained. A comparison of *rbc*L sequences between *P. heteroplatos* and *G. crinale* is shown in Table S1. *Pterocladia heteroplatos* was identical to *G. crinale* from Australasia, Spain, and Tanzania. In addition, full sequences of both COI-5P and *rbc*L from the isotype of *P. heteroplatos* (PC 0452740; labeled as “cotype”) were obtained and nested in *G. crinale* (Figure 1, C33; Figure S1, R25).

The ML tree of the 33 COI-5P haplotypes (Table S6) resolved five major groups (I–V) within *G. crinale* (Figure 1A): Group I (70% MLBS, 0.97 BPP) from the Atlantic and the Mediterranean Sea comprising east and west Atlantic subgroups; Group II, a singleton, from the Ionian Sea; Group III (70% MLBS, 1 BPP) from Asia, including Northeast and Southeast Asian subgroups; Group IV from Italy and Slovenia; Group V from Australasia, India, Easter Island (Chile), and Tanzania. The split network was consistent with those of the ML phylogeny, which also identified five groups (Figure 1B). The uncorrected pairwise distances among the five groups varied from 0.8% to 3.6% (Table S2). The pairwise distances within groups ranged from 0.2% to 3.0%.

The *rbc*L phylogeny (Figure S1) generally corresponded to those from the COI-5P, consisting of five groups with higher support in group IV (89% MLBS, 0.99 BPP) and V (79% MLBS, 1 BPP). The uncorrected pairwise distances among groups varied from 0.2% to 1.4% (Table S2). The pairwise distances within groups ranged from 0.08% to 1.1%.

Divergence time estimates using COI-5P sequences suggested that the split of *G. crinale* from its closest relatives, *G. americanum* and *G. calidum*, happened at approximately 5.94 Ma (95% highest posterior density [HPD] = 8.63–3.62 Ma) (Figure S2). The estimated age of the crown node of *Gelidium crinale* was 2.26 Ma (95% HPD = 3.19–1.44 Ma). The divergence of group I was traced back to about 0.99 Ma (95% HPD = 1.48–0.54 Ma), and group III likely diverged about 0.87 Ma (95% HPD = 1.35–0.44 Ma).

### 2.2. Genetic Diversity and Phylogeographic Structure Based on Mitochondrial COI-5P

The alignment of the COI-5P for 116 individuals was 495 nucleotides (nt) with 50 polymorphic sites (10%) including 35 parsimony informative sites (7%). Haplotype diversity (total *H*d = 0.933 ± 0.012) ranged from 0.000 to 1.000, and nucleotide diversity (total π = 0.0145 ± 0.0006) varied from 0.0000 to 0.0094 (Table 1). The Slovenia population had the highest haplotype and nucleotide diversity (*H*d = 1.000 ± 0.272, π = 0.0094 ± 0.0033). At a phylogroup level, most of the groups showed high genetics diversities (*H*d = 0.796–0.889, π = 0.0058–0.0133), except group IV (*H*d = 0.604, π = 0.0024). At the realm level, the Eastern Atlantic (44 specimens, *H*d = 0.712, π = 0.0087) and Asia (32 specimens, *H*d = 0.869, π = 0.0071) showed higher genetic diversity than those from the Western Atlantic (31 specimens, *H*d = 0.774, π = 0.0038) and Australasia (6 specimens, *H*d = 0.733, π = 0.0050).

**Figure 2:**
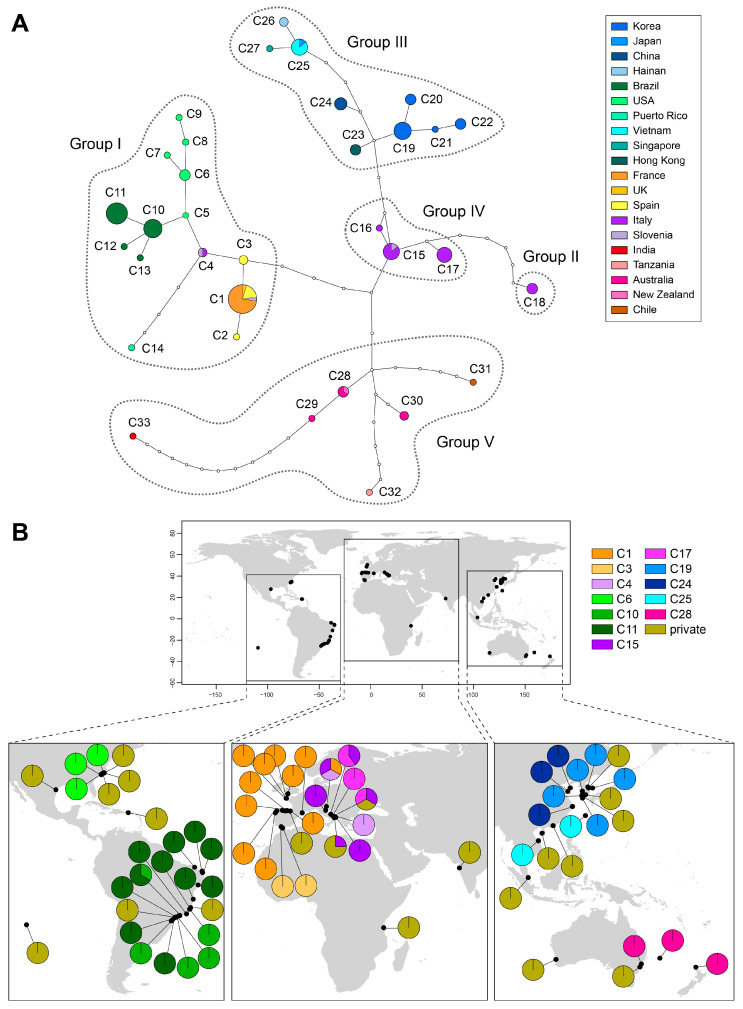
TCS-derived network and geographic distribution of 33 haplotypes of *G. crinale* based on mitochondrial COI-5P. (**A**) Haplotype network. Each circle denotes a single haplotype with size proportional to frequency. Small open circles represent missing haplotypes. Haplotypes are colored according to the country as shown in the key. Haplogroup follows phylogroups in Figure 1 and Figure S1. (**B**) Distribution map of haplotypes. Pie charts denote the proportion of haplotypes present in each location. Private and shared haplotypes are colored as shown in the key.

Thirty-three haplotypes were identified from 19 populations of which 28 haplotypes were restricted to a single population and five haplotypes were found in two to four populations (Figure 2). The TCS network revealed five geographical groups similar in COI-5P and *rbc*L phylogenies. These groups are represented by 4–7 bp changes from each other. The Atlantic-Mediterranean group (I), occurring on both sides of the Atlantic Ocean and the Mediterranean Sea, comprised four subgroups without any shared haplotype among them: (i) Brazil (C10-C13), (ii) Northeast America (C5-C9), (iii) Northeast Atlantic-Mediterranean (C1-C4), and (iv) Puerto Rico (C14). Each subgroup was connected by a single mutation, except C14 which was connected by three missing haplotypes from haplotype C4. The Northeast Atlantic-Mediterranean subgroup included the most common haplotype, C1, from France, the UK, and Spaand in Slovenia. The Adriatic-Ionian group (IV), comprising three haplotypes from 14 specimens, revealed clear evidence of genetic segregation in the Eastern Mediterranean Sea. The Ionian group (II, C18) included a single haplotype from a locality known as Cimino, in the Mar Piccolo of Taranto (southern Italy). It was segregated from group IV by six missing haplotypes.

The Asian group (III) included two subgroups; the Southeast Asian subgroup (C25–C27) comprised specimens from Hainan (China), Okinawa (Japan), Singapore, and Vietnam, and the Northeast Asian subgroup (C19–C24) found in Korea, China, and Hong Kong. Interestingly, haplotype C23 from Hong Kong was more closely related to the northeast haplotypes than those from Southeast Asia. East (C28 and C29) and west (C30) Australasian haplotypes differed by three missing haplotypes. Haplotypes from Easter Island (Chile; C31), India (C33), and Tanzania (C32) were connected with the Australasian haplotypes by five to ten missing haplotypes.

The gene diversity among populations (*H*_T_ = 0.978) was higher than that within populations (*H*_S_ = 0.535) (Table S3). A permutation test showed that *N*_ST_ was significantly higher than *G*_ST_ (*N*_ST_ = 0.726 > *G*_ST_ = 0.453; *p* < 0.01), indicating that *G. crinale* has a significant phylogeographical structure. Phylogroup and realm-level showed statistically significant phylogeographical structure (*N*_ST_ = 0.544 > *G*_ST_ = 0.368, *p* < 0.05; *N*_ST_ = 0.573 > *G*_ST_ = 0.228, *p* < 0.01).

Pairwise *F*_ST_ estimates exhibited high levels of genetic differentiation among five groups (Table 2). The highest difference was observed between groups II and IV (0.8600), while the lowest differentiation was observed between groups III and V (0.5140). At the realm level, the Western Atlantic considerably differed from the other realms (Table S4). The highest difference was observed between the Western Atlantic and the Australasia (0.8273), while the lowest differentiation was observed between the Australasia and the Western Indo-Pacific (0.4356). The non-significant differentiation between Eastern Indo-Pacific and others was likely due to low sampling. Population-level *F*_ST_ values also revealed high levels of genetic differentiation and limited connection among populations (Table S5). Three populations (France, Hong Kong, and Vietnam) which are geographically distant were highly differentiated (*F*_ST_ = 1.0000). The geographically proximate populations in Europe (France and Spain) were less differentiated (*F*_ST_ = 0.2667).

Nonhierarchical AMOVA (Table 3) showed that 82.5% of the genetic variation was found among populations (*p* < 0.001). A smaller but significant amount of genetic variation was found within populations (17.5%, *p* < 0.001). The hierarchical AMOVA showed that 46.2% (realm level)–54.7% (phylogroup level) of variation occurred among groups, followed by 35.7% (phylogroup level)-37.4% (realm level) variations among populations within groups (*p* < 0.001).

### 2.3. Historical Demography Inferences Based on Mitochondrial COI-5P

The Tajima’s *D* and Fu’s *F*_S_ values were statistically non-significant, negative at species level (*D* = −0.732, *F*_S_ = −6.520) and group I (*D* = −0.345, *F*_S_ = −3.007). Fu’s *F*_S_ value was significantly negative (*p* < 0.05) in the USA population (*F*_S_ = −2.477) and the Western Atlantic realm (*F*_S_ = −3.397), suggesting those populations’ expansion (Table 1).

**Figure 3:**
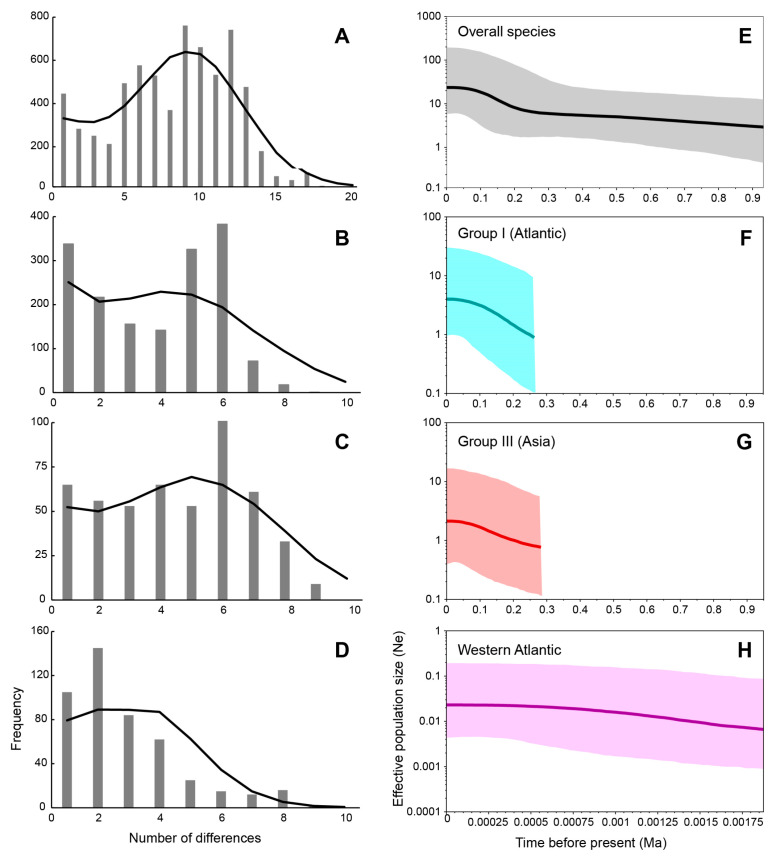
Mismatch distribution analysis (MDA) and Bayesian skyline plot (BSP) of *Gelidium crinale* using a mitochondrial COI-5P dataset. (**A**) MDA of overall species; (**B**) MDA of group I; (**C**) MDA of group III; (**D**) MDA of the Western Atlantic realm; (**E**) BSP of overall species; (**F**) BSP of group I; (**G**) BSP of group III; (**H**) BSP of the Western Atlantic realm. For mismatch distributions, the grey bar charts represent the observed distributions, whereas the black lines represent simulated data under a sudden expansion model. For BSPs, the *x*-axis represents the time since the present in years and the *y*-axis represents the estimated effective population size (*N*_e_). Light blue color indicates BSP for Group I, red for Group III, and pink for the Western Atlantic. The solid center line is the median estimate, and the upper and lower lines indicate the 95% highest posterior density (HPD) intervals for *N*_e_.

In mismatch distribution analyses (Figure 3A–D), the low and non-significant sum of squared deviation (SSD) and Harpending’s raggedness index (Hri) values indicated a good fit of the observed mismatch distribution from that obtained via model simulation under sudden demographic expansion. The multimodal distribution (Figure 3A,C) and neutrality tests of *G. crinale* (all populations) and group III revealed the population equilibrium. Group I (Figure 3B) showed bimodal distribution as a result of the presence of haplotypes of two subgroups: the first peak corresponding to intra-subgroup differences and the second peak corresponding to inter-subgroup differences. The Western Atlantic realm exhibited an observed unimodal mismatch frequency distribution (Figure 3D), fitting a recent sudden population expansion model.

The Bayesian skyline plots (BSP) (Figure 3E–G) depicted a model of the past increase of effective population size in the total population of the species (about 0.2 Ma), group I (about 0.25 Ma), and group III (about 0.25 Ma). However, this model was not revealed in the Western Atlantic realm (Figure 3H), which likely suffered a short coalescent time with a stable population size. The BSP for the USA population was not constructed because of a few variations accounting for a short coalescent time.

## 3. Discussion

Phylogenies of COI-5P and *rbc*L sequences support well the monophyly of *G. crinale* and its separation from *G. americanum* and *G. calidum*. The pairwise divergence of COI-5P (0.2–3.6%) within the species is similar to that (0.7–3.4%) of *Gelidiella fanii* S.-M.Lin, a cosmopolitan species [26]. In addition, our study demonstrates the occurrence of *G. crinale* in Easter Island, India, and Tanzania, as well as many locations in the Atlantic and Pacific Ocean regions. However, published COI-5P sequences of *G. crinale* from Hawaii match those of *G. sclerophyllum* [30], and further sampling is needed to confirm the previous report in Hawaii [20].

### 3.1. Genetic Diversity and Geographical Structure

Our study revealed that *Gelidium crinale* is structured into five main groups representing non-overlapping geographical regions. The Atlantic-Mediterranean group is the largest in the number of specimens analyzed, with its range covering from Brazil to North Carolina, USA, on the western Atlantic, and from Italy to England on the eastern Atlantic, including the Mediterranean Sea. One notable result is a continuous distribution of haplotype C1 from the Eastern Atlantic (England, northern France, and Spain) to the Mediterranean Sea (southern France, Italy, and Slovenia), suggesting northern expansion from the warm Mediterranean population because *G. crinale* occurs more commonly in warm waters than cold waters. However, a disjunct distribution of *Flabellia petiolata* (Turra) Nizamuddin, a green alga in Halimedaceae, between southern England and the Mediterranean Sea, is considered a relict population that once occurred probably during warmer periods (about 11,700 years ago) [31].

The Ionian group is distantly related to the Adriatic and Atlantic-Mediterranean populations. Considering that Taranto is a well-known location of eutrophication and introduced species in Italy [32], the Ionian group has likely been acclimated to eutrophication. Northern Mediterranean populations provide clear evidence of genetic fragmentation between the Adriatic and Ionian Seas, suggesting that the Adriatic Sea area likely served as an independent refugium.

The Asian group tends to have latitudinal segregation: the Southeast Asian subgroup is likely acclimated to tropical and subtropical waters, while the Northeast Asian subgroup is likely acclimated to temperate water, except the Hong Kong population. The border of the two lineages roughly matches the summer 28 °C and the winter 20 °C isotherms, suggesting that the range of these two lineages can be used as an indicator of northern expansion. This result is consistent with the segregation between *Gelidiophycus divaricatus* and *G. freshwateri* [27].

Southern Indo-Pacific groups revealed the most wide-ranged distribution with a high genetic divergence. The Indian specimen (as *Pterocladia heteroplatos*) is included in this lineage. Five subgroups were identified: (i) eastern Australia-New Zealand, (ii) western Australia, (iii) India, (iv) Tanzania, and (v) Easter Island (Chile). Despite their occupying similar temperature zones, east-west segregation in Australia is common in algal flora [33]. A shared haplotype between eastern Australia and New Zealand may indicate an old relict, as reported by Nelson and Farr [12]. We caution that our estimates must be interpreted with care due to the small sampling in these regions. Further study is needed to decipher the relationships of Indian, Tanzanian, and Easter Island taxa.

### 3.2. Pleistocene Relicts for Cosmopolitan Distribution

Using COI-5P substitution rate of florideophycean red algae [34], the origin of *G. crinale* was dated 8.63–3.62 Ma from the late Miocene to the Early Pliocene. In this period, surface seawater temperature was 2–3 °C higher than today’s ocean temperatures [35]. Differentiation between northern and southern hemisphere populations was dated to occur 2.63–1.26 Ma, approximately matching the Early Pleistocene. Simultaneously, the Asian lineage was likely differentiated from the Atlantic lineage 2.21–1.04 Ma. The BSP suggested a slow growth of the whole *G. crinale* population, approximately 0.2 Ma, and both the Atlantic-Mediterranean and Asian lineages revealed population growth approximately 0.25 Ma, all predating or nearly matching the onset (22–18 kya) of the Northern Hemisphere deglaciation [36]. It is inferred that *G. crinale* likely made northward and southward dispersals from the tropical region, then was recolonized by repeated range expansion in the temperate refugia where it currently occurs. *Gelidium crinale* likely made a pre-LGM expansion, as did *Palmaria palmata* (L.) F.Weber and D.Mohr, *Mazzaella laminarioides* (Bory) Fredericq, *Gelidiophycus divaricatus* (G.Martens) G.H.Boo, J.K.Park and S.M.Boo, and *G. freshwateri* G.H.Boo, J.K.Park and S.M.Boo [27,37,38]. However, many other seaweeds had post-LGM recolonization on both Atlantic coasts [39]. Because of the lack of a fossil record for *Gelidium,* as well as for most red algae [40,41,42], events of population expansion and lineage divergence in *G. crinale* need cautious interpretation.

*Gelidium crinale* has not been included in the recent lists of introduced or non-indigenous marine species elsewhere [32,43] except in New Zealand [44]. It was first reported as *Fucus crinalis* Hare *ex* Turner from England [45] before its name was changed to *Gelidium crinale* [46]. Afterward, it was reported in the Mediterranean Sea [47] and New Zealand (as *G. longipes*, [48]), then widely in the world. The records of *G. crinale* in Europe and New Zealand earlier than the 20th century are not in favor of a hypothesis of biological invasion, which primarily results from the twentieth century’s marine trade.

Our findings of geographical structure, the presence of lineage-specific private haplotypes, and the absence of shared haplotypes between lineages suggested that the cosmopolitan distribution of *G. crinale* has been shaped by relict populations, which survived prior to LGM. *Gelidium crinale* might have likely dispersed by sporadic events such as drifting, rafting on animals such as marine turtles (e.g., *Chelonia mydas*), and other modes of dispersal of benthic organisms without a planktonic phase [49]. Turfs or clumps detached by catastrophic events such as strong winds or tsunamis happened to move in a long distance and successfully settle down in favorable habitats as founder populations. The high capacity of regeneration of *G. crinale* [10,12] might have allowed its long-distance dispersal and colonization success in remote locations.

The lack of shared haplotypes between lineages of *G. crinale* does not support a hypothesis of recent introduction. If *G. crinale* was introduced from its origin center to elsewhere, an identical haplotype should be found [50]. Our results also did not reveal any mixtures of phylogenies or haplotype networks of alien populations, which are common in introduced alien species [51,52]. The COI-5P sequences from four specimens collected from Taranto in the Ionian Sea, a well-known location of introduced species in Italy [32], revealed two haplotypes, one private and the other shared, in the Adriatic Sea that are not found outside the Adriatic Sea. Although identical haplotypes occurred between the Ionian Sea the and Adriatic Sea or between Eastern Atlantic and the Mediterranean Sea, our genetic data did not reveal any signal on the anthropogenic introduction of *G. crinale* in multi-ocean regions.

### 3.3. Ecological Implication

Our study proposes intriguing issues about how such small-sized, wiry, turf-forming red alga has retained cosmopolitan distribution under extremely changeable climatic conditions during the interglacial period of the Pleistocene. Our study confirmed the occurrence of *G. crinale* at a river estuary in Jeoncheon, Korea, which has large salinity fluctuations. *Gelidium crinale* occurred abundantly throughout the year in the Port Aransas estuary with its relatively large temperature and salinity fluctuations [53]. A year-round domination of lower littoral populations from a North Carolina coastal jetty suggested that they have been acclimated to fluctuating seasonal environmental conditions [9]. *Gelidium crinale*, occurring year-round in the Patos Lagoon estuary, Brazil, revealed a positive growth response to elevated salinities, indicating its euryhaline tolerance [54]. *Gelidium crinale* from Israel revealed a maximum growth rate at a mean water temperature of 29.3 °C and high light intensity and high ammonium concentrations [55]. *Gelidium crinale* is thus considered sciophilous, a designation also supported by its burial beneath sand during warm seasons [10]. *Gelidium crinale* also accumulated heavy metals (Cd, 0.54 μg/g dry weight, Cu, 9.40 μg/g dry weight, and Fe 410 μg/g dry weight), being similar to or higher than those of other seaweeds from Marsa-Matrouh beaches, Egypt [56]. The genetic isolation of specimens from the Mar Piccolo of Taranto in the present study suggests that they are likely acclimated to moderate eutrophication. This basin is, on average, considered mesotrophic [57], but the locality of specimen collections (Cimino) is in a zone with an active sewage outfall and a mussel farming area [58]. Taken together, global populations of *G. crinale* may have retained certain strategies to be tolerant or susceptible to various environmental stresses of temperature, salinity, irradiance, nutrients, heavy metal, and eutrophication.

Considering that gametophytes only occurred in about 6% of the total collection from Portugal [11] during sporadic dispersal events, spreading almost certainly depends on fragmentation and a high capacity for regeneration of vegetative fragments or asexual tetrasporophytes. This behavior in *G. crinale* further increases the survival and establishment success of rafters, as reported in *Pterocladiella* [42].

*Gelidium crinale* might be dispersed by actively moving grazers likely during the Pleistocene. For example, plants of *G. crinale* are reported to be diets for juvenile green turtles [59] and act as nurseries for various invertebrates, such as crustacea and molluscans [60].

### 3.4. Taxonomic Implication

**Figure 4:**
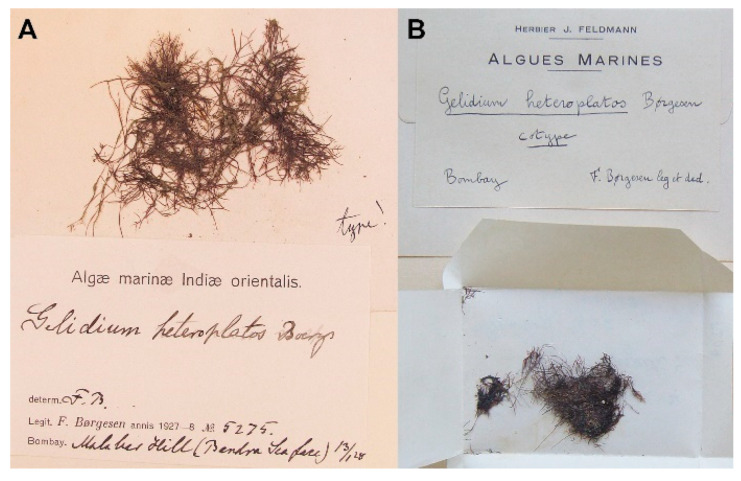
Type specimens of *P. heteroplatos* analyzed in this study. (**A**): Holotype specimen (*Børgesen 5275* in C); (**B**): Isotype specimen (PC 0452740, labelled as ‘cotype’).

Our analysis of mitochondrial COI-5P and plastid *rbc*L sequences from the holotype (*Børgesen 5275* in C; herbarium code followed by Thiers [61]) and the isotype (PC 0452740 in PC) materials of *G. heteroplatos* was consistently nested in *G. crinale*. Our result does not support the transfer of *G. heteroplatos* to the genus *Pterocladia*. We suggest that *G. heteroplatos* from Malabar Hill, Mumbai (Bombay), India (Figure 4), is likely different from the taxon used for the revision of *Pterocladia heteroplatos* [21]. First, the material used for the taxonomic revision was not from the type locality but from Visakahapanatum in eastern India. Second, the habit and unilateral branching of a cystocarpic plant (see Figure 1c in [21]) does not match the illustration of the type (see Figure 3 in [19]). Further DNA analysis needs to confirm whether the eastern Indian taxon, named *Pterocladia heteroplatos*, is a misidentification of another species or a new species. Its reports in Australia, East Africa, the Red Sea, and the Southeast Asian regions likely result from a misidentification of *G. crinale*.

*Gelidium crinale* includes several infraspecific taxa from the Mediterranean Sea [62], Eritrea [63], Japan [64], and the USA [65]. *Gelidium crinale* is quite variable in habit, as shown in images of specimens from Korea, Hong Kong, and Spain (see Figure 4A–D in [8]). Our sampling included topotypes or specimens near the type locality of those infraspecific taxa, with the exception of var. *perpusillum*. However, any genetic groups of our COI-5P and/or *rbc*L trees are not distinct enough to keep the infraspecific taxa, as was reported by Kim and Boo [8]. Although Boo et al. [18] reported subsp. *longipes* based on geographical structure of Australasian specimens, the present taxon-wide analysis also does not support its infraspecific classification. We propose that *G. crinale* is a single species across multi-ocean regions, and its infraspecific subdivision could not be supported.

#### Taxonomic Conclusion

On the basis of sequences from type materials, here, we merge *Gelidium heteroplatos* with *G. crinale*.

*Gelidium crinale* (Hare ex Turner) Gaillon 1828 (p. 362)

Heterotypic synonyms: *Gelidium heteroplatos* Børgesen (Kew Bull. 1:3, Figure 3. 1934); *Pterocladia heteroplatos* (Børgesen) Umamaheswara Rao and Kaliaperumal (Bull. Bot. Surv. India 22: 109, Figure 1. 1980).

## 4. Materials and Methods

### 4.1. Sampling and Herbarium Collections

**Figure 5:**
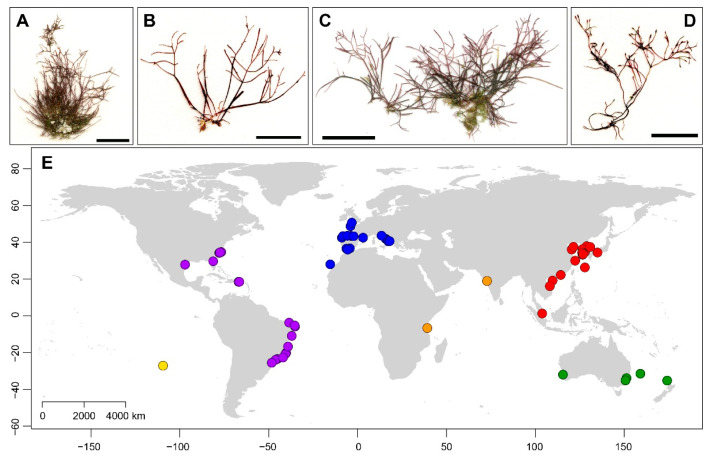
Habit (**A**–**D**) of representative specimens and map (**E**) of collection sites of *Gelidium crinale*. (**A**): France; (**B**): England; (**C**): Vietnam; (**D**): Korea. Color dots indicate six biogeographic realms (blue, Eastern Atlantic; purple, Western Atlantic; red, Asia; orange, Western Indo-Pacific; green, Temperate Australasia; yellow, Eastern Indo-Pacific).

Fresh materials were collected from Australia, Chile, China, France, Italy, Japan, Korea, Singapore, Slovenia, Spain, and Vietnam (Figure 5; Table S6). They were air-dried on a lab bench for 1–2 days and preserved in silica gel for molecular analysis. A specimen from Tanzania was obtained at the macroalgal herbarium, Ghent University, Belgium (GENT). We obtained fragments (approximately 5 mm in size) from the holotype and an isotype (labeled as “cotype”) of *Pterocladia heteroplatos* from India, with the permission of curators at the Natural History Museum at the University of Copenhagen, Denmark (C), and the cryptogam collection of the Muséum National d’Histoire Naturelle in Paris, France (PC). Information on all samples used in the present study and publicly released sequences from GenBank are given in Supplementary Table S6.

### 4.2. DNA Extraction, PCR, and Sequencing

DNA extraction, amplification, and sequencing of both COI-5P and *rbc*L followed Boo et al. [25]. The primers used for amplifying and sequencing were F7, F645, R753, and RrbcS-start for *rbc*L [23,66,67], and COXI43F and COXI1549R for *cox*1 [68]. We first analyzed COI-5P from fresh and herbarium specimens, then analyzed *rbc*L from representative specimens of COI-5P lineages. The total DNA dataset contained 163 sequences of *G. crinale*, including previously published 62 COI-5P and 42 *rbc*L sequences from GenBank. All sequences were aligned using the MUSCLE algorithm in MEGA7 [69] with default parameters, and the alignment was manually adjusted. *Gelidium americanum* (W.R.Taylor) Santelices and *G. calidum* Jamas, Iha and Fujii were used as outgroups on their close relationship [70,71].

### 4.3. DNA Extraction and High Throughput Sequencing of Type Specimens of P. heteroplatos

DNA damages of plant herbarium specimens are challenging [72]. DNA was extracted from holotype material (*Børgesen 5275*) of *Pterocladia heteroplatos* with strict adherence to the precautionary guidelines outlined by Hughey and Gabrielson [73] using the DNeasy Blood and Tissue Kit (Qiagen, Valencia, CA, USA). DNA is typically highly degraded; a short *rbc*L region (124 bp) was analyzed using primers F753 and R900 [74]. Sequencing was performed by Genotech Co. (Daejeon, Korea).

The high-throughput sequencing of the isotype material of *P. heteroplatos* (PC 0452740) was performed by High Throughput Genomics Unit, University of Washington (UW-HTGU, Seattle, WA, USA). The 36 bp paired-end sequencing analysis was performed by UW-HTGU using the manufacturer’s protocol via the cBot and HiSeq 2000 (Illumina, San Diego, USA). The adapters and low-quality reads were eliminated using Trimmomatic v0.39 [75]. The COI-5P and *rbc*L sequences were extracted by mapping reads onto reference sequences of *G. crinale* using the mem algorithm of BWA 0.7.17 [76] with default settings.

### 4.4. Phylogenetic Analysis

Phylogenies of mitochondrial COI-5P and plastid *rbc*L datasets were reconstructed using maximum likelihood (ML) and Bayesian inference (BI). The ML analyses were performed using the Pthreads version of RAxML v8.0.X [77] set as follows: a rapid bootstrap analysis and search for the best-scoring ML tree in one single run with 1000 bootstrap (BS) replicates under the GTRGAMMA model. The BI analyses were performed for individual datasets with MrBayes v3.2.1 [78] using the Metropolis-coupled Markov Chain Monte Carlo (MC3) with the best-fitting substitution model. The best-fitting nucleotide substitution model was selected using Modeltest v3.7 [79] with Akaike Information Criteria (AIC). For each matrix, two million generations of two independent runs were performed with four chains and sampling trees every 100 generations. Twenty-five percent of saved trees were removed as burn-in, and the remaining trees were used to infer Bayesian posterior probabilities (BPP). A split network was constructed in SplitsTree5 [80], which detects reticulation signals, using the Neighbor-Net method under the Kimura-2-parameter distance model.

### 4.5. Divergence Time Estimation

BEAST v.1.8.1 [81] was used to estimate the divergence time based on the mitochondrial COI-5P sequences. No fossils were available to calibrate the nodes. Therefore, a controversial substitution rate of 0.76%/Ma [34] was proposed and the best fit HKY + I + G model was selected in the present study. Data were analyzed using a Birth Death Process tree prior [82] with a strict clock model. Two independent MCMC analyses of 20 million generations were performed with tree sampling every 1000 generations to obtain posterior distributions of parameters. The convergence of each analysis was determined in Tracer v.1.7 [83], examining the effective sampling size (ESS) for all parameters. The ESS was >200 for all parameters. The maximum clade credibility (MCC) tree was generated with TreeAnnotator v.1.8.1 after discarding 10% of the saved trees as burn-in.

### 4.6. Phylogeographic Analysis of Mitochondrial COI-5P

We inferred the genetic relationships between haplotypes using the network estimation approach under 95% probability criterion for a parsimonious connection, as implemented in TCS [84]. The network was graphically visualized using tcsBU [85]. Haplotype diversity (*h*) and nucleotide diversity (*π*) were calculated for each population (*h*_S_ and π_S_) and at the species level (*h*_T_ and π_T_) using DnaSP v.6 [86]. Population diversity indices were estimated using the software PERMUT [87]. The within-population diversity (*h*_S_), total diversity (*h*_T_), geographical average haplotype diversity (*v*_S_), geographical total haplotype diversity (*v*_T_), level of population differentiation at the species level (*G*_ST_), and an estimate of population subdivisions for phylogenetically ordered alleles (*N*_ST_) were calculated.

The occurrence of significant phylogeographic structure was inferred by testing whether *G*_ST_ (the index that considers only haplotype identities) and *N*_ST_ (the index that takes into account a measure of haplotype divergence) were significantly different using 1000 permutations in PERMUT [87]. Non-hierarchical and hierarchical analyses of molecular variance (AMOVA) were performed using Arlequin v.3.5 [88] with Φ-statistics to quantify the proportion of total genetic variance, with significance of fixation indices tested using 10,000 permutations.

### 4.7. Demographic History

Historical demography was inferred using the COI-5P dataset. The null hypotheses of spatial expansion and pure demographic expansion, respectively, were tested using mismatch distribution analysis (MDA) in Arlequin v.3.5. For each expansion model, goodness-of-fit was tested with the sum of squared deviations (SSD) and Harpending’s raggedness index (*H*_Rag_) using 1000 parametric bootstrap replicates [89]. We also performed tests of selective neutrality (Tajima’s *D* and Fu’s *F_S_*) to infer potential population growth and expansion [90,91].

BEAST v.1.8.1 [81] was used to infer the demographic histories by constructing Bayesian skyline plots (BSPs) of effective population size (*N_e_*) through time [92]. According to Modeltest v.3.7 [79], the HKY + I, TrN, and HKY models were selected for overall species, groups I and III, respectively. The same substitution rate (0.76% per Ma) used in MDA was also adopted here. The Markov chain Monte Carlo (MCMC) was run for 1 × 10^7^ generations with trees sampled every 1000 generations and the first 10% of the samples were discarded as burn-in. The result was visualized by Tracer v.1.7 [83]. Three replicate runs using different random seeds were conducted to confirm convergence.

## 5. Conclusions

Both COI-5P and *rbc*L sequence analyses highlight a single origin of *Gelidium crinale* despite its cosmopolitan distribution in the world’s oceans and its morphological variability from different regions. Our study extends its range to India (as *Pterocladia heteroplatos*), Tanzania, and Easter Island. The main findings are the presence of five geographical lineages, population expansion via long-distance dispersal prior to LGM, and current distribution has been shaped by relict populations. A brief view reveals that the survival of *G. crinale* in environmentally heterogeneous regions may be attributed to its tolerance under various environmental stresses, including eutrophication. Our study serves as an example of the exceptional cosmopolitan distribution of the small-sized turf alga. A phylogenomic approach with additional sampling in understudied regions such as the Red Sea and the Eastern Indo-Pacific may overcome statistical limitations for inference of the phylogeography of *G. crinale*.

## Figures and Tables

**Table 1:** Information of sample sizes and genetic characteristics of 19 populations, five phylogroups, and six realms of *Gelidium crianle* based on mitochondrial COI-5P sequences.

Population	n	S	nh	π ± SD	Hd ± SD	Tajima’s *D*	Fu’s *F_S_*
Australia	5	5	3	0.0057 ± 0.0014	0.800 ± 0.164	1.124	1.220
Brazil	23	3	4	0.0014 ± 0.0002	0.597 ± 0.063	−0.368	−0.694
Chile	1	NA	1	NA	NA	NA	NA
China	6	5	2	0.0054 ± 0.0017	0.533 ± 0.172	1.219	3.696
France	16	0	1	0.0000 ± 0.0000	0.000 ± 0.000	NA	NA
Hong Kong	3	0	1	0.0000 ± 0.0000	0.000 ± 0.000	NA	NA
India	1	NA	1	NA	NA	NA	NA
Italy	17	13	5	0.0073 ± 0.0016	0.757 ± 0.063	−0.239	2.156
Japan	1	NA	1	NA	NA	NA	NA
Korea	15	3	4	0.0022 ± 0.0005	0.676 ± 0.101	0.587	−0.116
New Zealand	1	NA	1	NA	NA	NA	NA
Puerto Rico	1	NA	1	NA	NA	NA	NA
Singapore	1	NA	1	NA	NA	NA	NA
Slovenia	3	7	3	0.0094 ± 0.0033	1.000 ± 0.272	NA	0.308
Spain	7	2	3	0.0015 ± 0.0005	0.667 ± 0.160	−0.275	−0.438
Tanzania	1	NA	1	NA	NA	NA	NA
UK	1	NA	1	NA	NA	NA	NA
USA	7	4	5	0.0027 ± 0.0007	0.857 ± 0. 137	−0.876	−2.477 *
Vietnam	6	0	1	0.0000 ± 0.0000	0.000 ± 0.000	NA	NA
Overall	116	50	33	0.0145 ± 0.0006	0.933 ± 0.012	−0.732	−6.520
**Phylogenetic groups**							
Group I	58	15	14	0.0058 ± 0.0003	0.796 ± 0.038	−0.345	−3.007
Group II	3	0	1	0.0000 ± 0.0000	0.000 ± 0.000	NA	NA
Group III	32	12	9	0.0071 ± 0.0005	0.869 ± 0.031	0.577	0.021
Group IV	14	3	3	0.0024 ± 0.0003	0.604 ± 0.076	0.826	1.252
Group V	9	23	6	0.0133 ± 0.0035	0.889 ± 0.091	−1.100	0.607
**6 realms**							
Eastern Atlantic	44	15	8	0.0087 ± 0.0010	0.712 ± 0.061	0.762	2.346
Western Atlantic	31	12	10	0.0038 ± 0.0008	0.774 ± 0.055	−1.231	−3.397 *
Asia	32	12	9	0.0071 ± 0.0005	0.869 ± 0.031	0.577	0.021
Australasia	6	5	3	0.0050 ± 0.0014	0.733 ± 0.155	0.708	1.420
Western Indo-Pacific	2	15	2	0.0303 ± 0.0152	1.000 ± 0.500	NA	2.708
Eastern Indo-Pacific	1	NA	1	NA	NA	NA	NA

n, number of analyzed samples; S, number of variable sites; nh, number of haplotypes; π, nucleotide diversity; Hd, haplotype diversity; SD, standard deviation; NA, not applicable; * *p* < 0.05.

**Table 2:** Pairwise *F*_ST_ comparison between five phylogroups of *G. crinale*.

	1	2	3	4	5
1. Group I	–				
2. Group II	**0.7515**	–			
3. Group III	**0.7020**	**0.6966**	–		
4. Group IV	**0.6854**	**0.8600**	**0.5812**	–	
5. Group V	**0.6815**	**0.5769**	**0.5140**	**0.5258**	–

Bold indicates *p* < 0.05.

**Table 3:** Analyses of molecular variance (AMOVA) of mitochondrial COI-5P sequences.

Grouping	Source of Variation	d.f.	Sum of Squares	Variance Components	Percentage of Variation	*F*-Statistics
non-hierarchical	Among populations	18	353.788	3.3152	82.49	**0.8249**
	Within populations	97	68.241	0.7035	17.51	
5 phylogroups	Among groups	4	249.520	2.5789	54.71	**0.5471**
	Among populations within groups	17	129.851	1.6808	35.66	**0.7874**
	Within populations	94	42.659	0.4538	9.63	**0.9037**
4 realms *	Among groups	3	208.992	1.9763	46.15	**0.4616**
	Among populations within groups	12	115.196	1.6020	37.41	**0.6949**
	Within populations	97	68.242	0.7035	16.43	**0.8357**

d.f.: degrees of freedom. Bold indicates *p* < 0.001. * The Western and Eastern Indo-Pacific realms were removed from the analysis because of the small number of samples.

## Data Availability

Not applicable.

## Supplementary Materials

The supporting information can be downloaded at: https://www.mdpi.com/article/10.3390/ijms24065263/s1.
