# Supplementary material for: Genetic Diversity and Phylogeography of a Turf-Forming Cosmopolitan Marine Alga, Gelidium crinale (Gelidiales, Rhodo-Phyta)
Source: Int J Mol Sci. 2023 Mar 9;24(6):5263. doi: 10.3390/ijms24065263 (PMC10049384; doi:10.3390/ijms24065263)
Supplement: Supplementary file 1 [file ijms-24-05263-s001.zip › BooEtAl_Gcrinale_phylogeography_suppl.pdf]

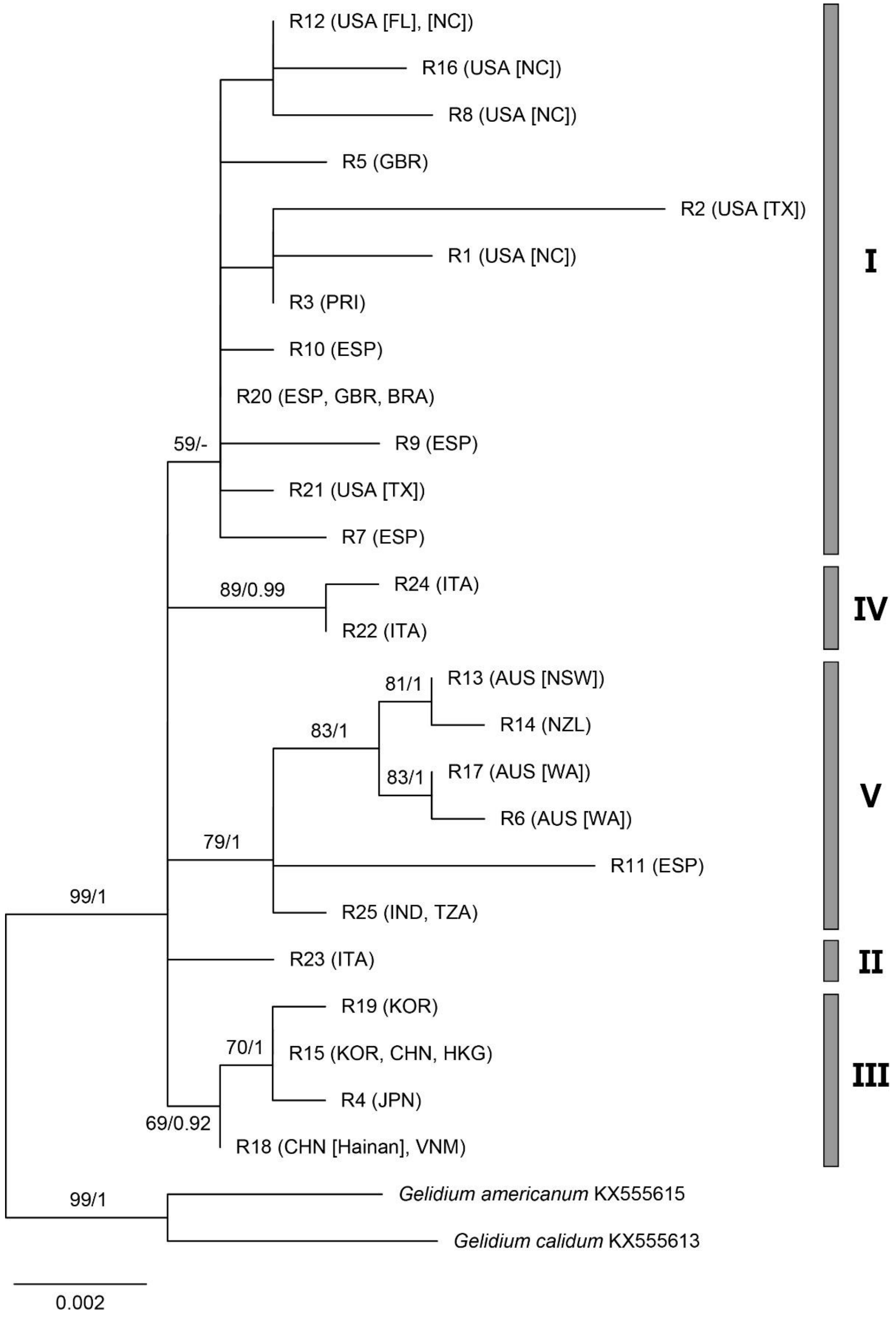

Figure S1. Maximum likelihood (ML) phylogeny of *Gelidium crinale* using plastid *rbc*L sequences. ML bootstrap values (≥50%) and Bayesian posterior probabilities (≥0.9) are shown at branches. Dash indicates values <50 or <0.9.

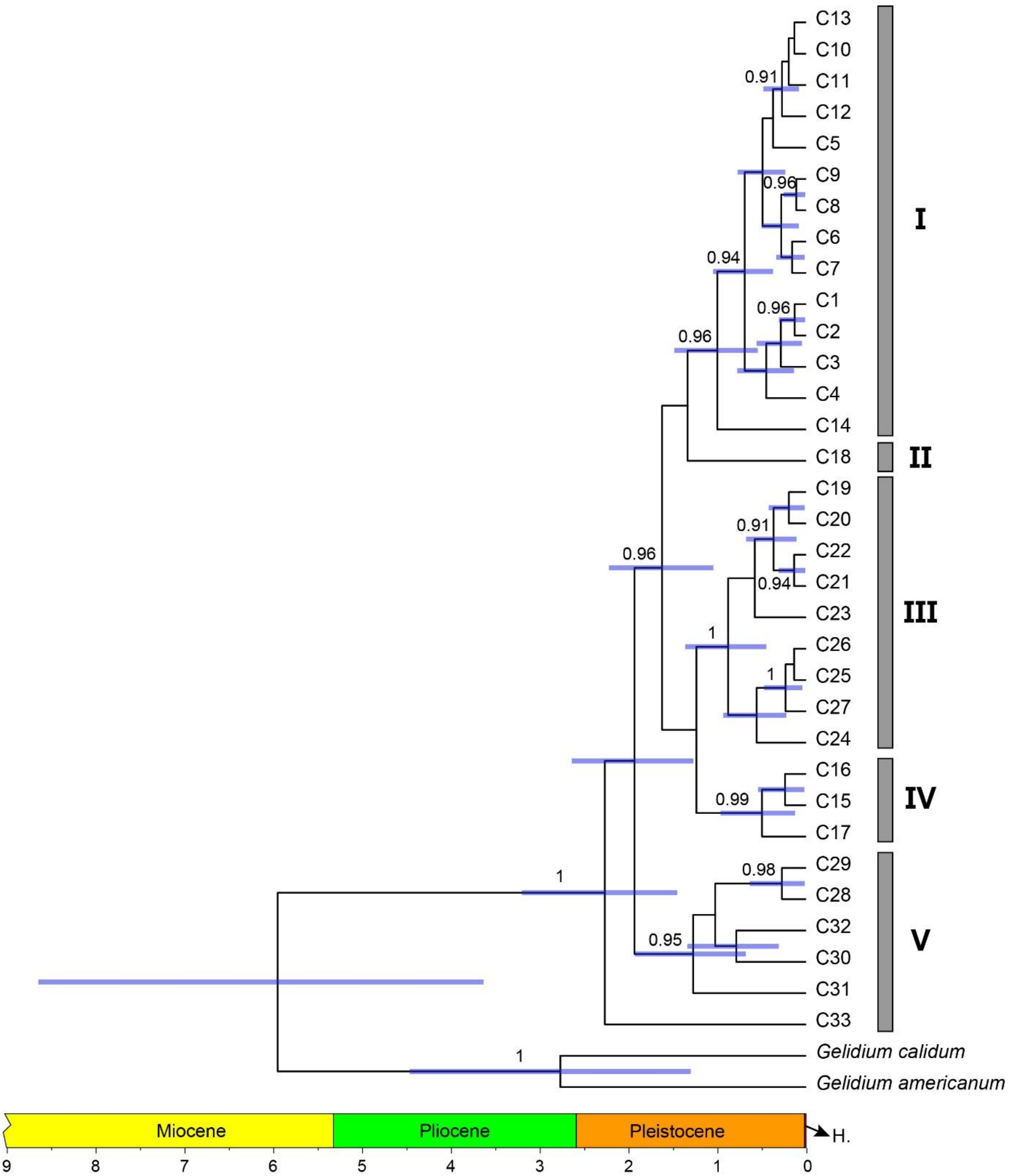

Figure S2. Time-calibrated phylogeny of *Gelidium crinale* based on COI-5P haplotypes. The time scale is printed in Ma. Blue bars represent 95% highest posterior density intervals. Numbers above or below branches are posterior probabilities (≥ 0.9 are shown).

Supplementary Table S1. Comparison of partial *rbc*L sequences (124 bp) at which base-pair variation occurred between *Gelidium crinale* and *Pterocladia heteroplatos* including two outgroups (*G. americanum* and *G. calidum*).

	**13**	**16**	**32**	**106**	**118**	**124**
Gelidium crinale-Group I (UK)	A	A	G	C	C	G
G. crinale-Group II (Italy)	•	•	•	•	T	•
G. crinale-Group III (Korea)	•	•	•	•	T	•
G. crinale-Group IV (Italy)	•	•	•	•	T	•
G. crinale-Group V (Western Australia)	•	•	•	•	T	T
G. crinale-Group V (New Zealand)	•	•	•	•	T	T
G. crinale-Group V (Spain)	•	•	•	•	T	T
G. crinale-Group V (Tanzania)	•	•	•	•	T	T
Pterocladia heteroplatos (holotype, *Børgesen 5275*)	•	•	•	•	T	T
Pterocladia heteroplatos (co-type, PC 0452740)	•	•	•	•	T	T
Gelidium americanum	•	•	A	T	T	•
Gelidium calidum	G	G	A	•	T	•

Supplementary Table S2. Pairwise divergence comparison of mitochondrial COI-5P and plastid *rbc*L sequences between five groups.

	**1**	**2**	**3**	**4**	**5**
1. Group 1	COI-5P: 0.2-1.6<br>*rbc*L: 0.08-1.1	0.2-0.9	0.2-1.0	0.3-1.1	0.3-1.4
2. Group 2	1.6-2.4	na	0.2-0.4	0.4-0.5	0.4-0.8
3. Group 3	1.4-2.6	1.8-2.2	0.2-1.6<br>0.08-0.2	0.3-0.6	0.3-0.9
4. Group 4	1.0-2.0	1.4-1.6	0.8-2.0	0.2-0.6<br>0.08	0.5-1.0
5. Group 5	1.4-3.6	1.8-3.0	1.0-3.2	0.8-2.8	0.2-3.0<br>0.08-0.8

Supplementary Table S3. Estimates of average gene diversity within populations (H_S), total gene diversity (H_T), interpopulation differentiation (G_{ST}) and number of substitution types (N_{ST}) for mitochondrial COI-5P haplotypes of *Gelidium crinale*.

	H_S	V_S	H_T	V_T	G_{ST}	N_{ST}
Population-level	0.535 (0.110)	0.274 (0.097)	0.978 (0.027)	1.000 (0.085)	0.453 (0.113)	0.726 (0.098)**
Phylogroups	0.632 (0.166)	0.473 (0.138)	1.000 (0.063)	1.038 (0.142)	0.368 (0.170)	0.544 (0.119)*
Realms	0.772 (0.035)	0.467 (0.116)	1.000 (0.024)	1.094 (0.030)	0.228 (0.039)	0.573 (0.148)**

Parentheses indicates standard error. * $p < 0.05$, ** $p < 0.01$.

Supplementary Table S4. Pairwise F_{ST} comparison between six realms of *G. crinale*.

	**1**	**2**	**3**	**4**	**5**	**6**
1. Eastern Atlantic	–					
2. Western Atlantic	**0.4676**	–				
3. Asia	**0.5353**	**0.7621**	–			
4. Temperate Australia	**0.5180**	**0.8273**	**0.5758**	–		
5. Western Indo-Pacific	**0.5900**	**0.8247**	**0.6366**	**0.4356**	–	
6. Eastern Indo-Pacific	0.6153	0.8656	0.6972	0.6410	-0.1567	–

Bold indicates $p < 0.05$.

Supplementary Table S5. Pairwise F_{ST} comparison between populations of *G. crinale*.

	1	2	3	4	5	6	7	8	9	10	11	12	13	14	15	16	17	18	19
1. France	–																		
2. Spain	**0.2667**																		
3. UK	0.0000	-0.7799																	
4. Italy	**0.7515**	**0.6382**	0.5152																
5. Slovenia	**0.6457**	0.1948	-0.7512	0.3008															
6. USA	**0.9098**	**0.7568**	0.7005	**0.5991**	**0.4662**														
7. Brazil	**0.9119**	**0.8414**	**0.8499**	**0.7697**	**0.7583**	**0.6972**													
8. Puerto Rico	1.0000	0.8718	1.0000	0.4987	0.1260	0.7946	0.8958												
9. Korea	**0.9391**	**0.8847**	0.8742	**0.6303**	**0.7635**	**0.8930**	**0.9253**	0.8741											
10. China	**0.9306**	**0.8361**	0.7366	**0.5712**	**0.6083**	**0.8329**	**0.9090**	0.7164	**0.6554**										
11. Japan	1.0000	0.9313	1.0000	0.5693	0.4849	0.8825	0.9412	1.0000	0.8067	0.1081									
12. Hong Kong	**1.0000**	**0.9290**	1.0000	**0.5229**	0.6503	**0.9047**	**0.9419**	1.0000	**0.6705**	**0.5586**	1.0000								
13. Singapore	1.0000	0.9371	1.0000	0.5658	0.5369	0.8707	0.9356	1.0000	0.8359	0.3324	1.0000	1.0000							
14. Vietnam	**1.0000**	**0.9626**	1.0000	**0.6985**	**0.8432**	**0.9362**	**0.9528**	1.0000	**0.8625**	**0.5541**	0.0000	**1.0000**	1.0000						
15. Australia	**0.9303**	**0.8206**	0.6910	**0.5149**	**0.5458**	**0.8313**	**0.9093**	0.6910	**0.7742**	**0.6689**	0.6972	**0.6917**	0.7276	**0.8639**					
16. New Zealand	1.0000	0.9040	1.0000	0.3884	0.3007	0.8742	0.9355	1.0000	0.8360	0.6681	1.0000	1.0000	1.0000	1.0000	-0.5582				
17. Tanzania	1.0000	0.9422	1.0000	0.6713	0.6053	0.9161	0.9568	1.0000	0.9069	0.7864	1.0000	1.0000	1.0000	1.0000	0.6129	1.0000			
18. India	1.0000	0.9532	1.0000	0.7345	0.6714	0.9204	0.95805	1.0000	0.9264	0.8211	1.0000	1.0000	1.0000	1.0000	0.7813	1.0000	1.0000		
19. Chile	1.0000	0.9370	1.0000	0.6383	0.5665	0.9081	0.9496	1.0000	0.8982	0.7806	1.0000	1.0000	1.0000	1.0000	0.6022	1.0000	1.0000	1.0000	–

Bold indicates $p < 0.05$.
