# Supplementary material for: Genetic Diversity and Phylogeography of a Turf-Forming Cosmopolitan Marine Alga, Gelidium crinale (Gelidiales, Rhodo-Phyta)
Source: Int J Mol Sci. 2023 Mar 9;24(6):5263. doi: 10.3390/ijms24065263 (PMC10049384; doi:10.3390/ijms24065263)
Supplement: Supplementary file 1 [file ijms-24-05263-s001.zip › TableS6_crinale_samplelist_rev.pdf]

Supplementary Table S6. List of published sequences for molecular analyses.

Species	Voucher-code	Collection site
Gelidium americanum	SP469081	Pedra do Sal, Luis Correia, Piauí, Brazil
Gelidium calidum	SP469077	Jauá Beach, Camaçari, Bahia, Brazil
Gelidium crinale	CNU056305	Gordon's Bay, Sydney, NSW, Australia
Gelidium crinale	LHI-1	Old Gulch, Lord Howe Island, NSW, Australia
Gelidium crinale	NSW-7	Summer Cloud Bay, Jervis Bay, NSW, Australia
Gelidium crinale	-	Fish Hook Bay, Rottnest Island, WA, Australia
Gelidium crinale	-	Fish Hook Bay, Rottnest Island, WA, Australia
Gelidium crinale	WA09	Green Island, Rottenest Island, WA, Australia
Gelidium crinale	WA12	Green Island, Rottenest Island, WA, Australia
Gelidium crinale	IBT0204	Brava Beach, Ubatuba, Sao Paulo, Brazil
Gelidium crinale	IBT0207	Brava Beach, Ubatuba, Sao Paulo, Brazil
Gelidium crinale	IBT0194	Brava Beach, Ubatuba, Sao Paulo, Brazil
Gelidium crinale	IBT0114	Saco da Ribeira, Ubatuba, Sao Paulo, Brazil
Gelidium crinale	IBT0109	Saco da Ribeira, Ubatuba, Sao Paulo, Brazil
Gelidium crinale	IBT0233	Domingas Dias Beach, Ubatuba, Sao Paulo, Brazil
Gelidium crinale	IBT0373	Cambriú Beach, Cardoso Island, Sao Paulo, Brazil
Gelidium crinale	IBT0273	Cibratel Beach, Itanhaém, Sao Paulo, Brazil
Gelidium crinale	IBT0362	Cibratel Beach, Itanhaém, Sao Paulo, Brazil
Gelidium crinale	IBT0272	Sonho Beach, Itanhaém, Sao Paulo, Brazil
Gelidium crinale	IBT0251	Éden Beach, Guarujá, Sao Paulo, Brazil
Gelidium crinale	IBT0250	Éden Beach, Guarujá, Sao Paulo, Brazil
Gelidium crinale	IBC0586	Enseada Azul, Meaípe, Espirito Santo, Brazil
Gelidium crinale	IBT0674	Manguinhos, Manguinhos, Espirito Santo, Brazil
Gelidium crinale	IBC0560	Parati Beach, Anchieta, Espirito Santo, Brazil
Gelidium crinale	IBC0559	Parati Beach, Anchieta, Espirito Santo, Brazil
Gelidium crinale	SP469067	Encantadas, Ilha do Mel, PR, Brazil
Gelidium crinale	SP469065	Espelho Beach, Porto Seguro, BA, Brazil
Gelidium crinale	SP469064	Aracaju, Barra dos Coqueiros, SE, Brazil
Gelidium crinale	SP469061	Munguba Beach, Paracuru, CE, Brazil
Gelidium crinale	IBT0903	Pai Vitório Beach, Armação de Búzios, Rio de Janeiro, Brazil
Gelidium crinale	SP469062	Rio do Fogo Beach, Maxaranguape, Rio Grande do Norte, Brazil
Gelidium crinale	IBT0276	Rio do Fogo Beach, Rio do Fogu, Rio Grande do Norte, Brazil
Gelidium crinale	EIS-023 (CNU065686)	La Perousse, Easter Island, Valparaiso, Chile
Gelidium crinale	CNU000179	Donghai road, Qingdao, China
Gelidium crinale	CNU000181	Donghai road, Qingdao, China
Gelidium crinale	P1575	Hainan, China
Gelidium crinale	P1576	Hainan, China
Gelidium crinale	CNU010865	Oriental Hotel, Yantai, China
Gelidium crinale	CNU065270	Putuoshan, Shanghai, China
Gelidium crinale	CNU065270-1	Putuoshan, Shanghai, China
Gelidium crinale	PC0171059 (LLG2997)	Lampaul île Ségal, Finistère, Brittany, France
Gelidium crinale	GHB0484	Fort Miradou, Collioure, Occitanie, France
Gelidium crinale	GHB0485	Fort Miradou, Collioure, Occitanie, France
Gelidium crinale	GHB0486-1	Fort Miradou, Collioure, Occitanie, France
Gelidium crinale	GHB0492	Fort Miradou, Collioure, Occitanie, France
Gelidium crinale	GHB0256	Ile Verte, Roscoff, Brittany, France
Gelidium crinale	GHB0260	Ile Verte, Roscoff, Brittany, France
Gelidium crinale	GHB0261	Ile Verte, Roscoff, Brittany, France
Gelidium crinale	GHB0265	Ile Verte, Roscoff, Brittany, France
Gelidium crinale	GHB0266	Ile Verte, Roscoff, Brittany, France
Gelidium crinale	GHB0269	Ile Verte, Roscoff, Brittany, France

Gelidium crinale	GHB0273	Ile Verte, Roscoff, Brittany, France
Gelidium crinale	GHB0337	Pointe de Perharidy, Roscoff, Brittany, France
Gelidium crinale	GHB0355	Pointe de Perharidy, Roscoff, Brittany, France
Gelidium crinale	GHB0357	Pointe de Perharidy, Roscoff, Brittany, France
Gelidium crinale	Hongkong21	Seoko, Hong Kong
Gelidium crinale	G2707	Seoko, Hong Kong
Gelidium crinale	G2710	Seoko, Hong Kong
Gelidium crinale	Battelli3	Koper Bay, Slovenia
Gelidium crinale	Battelli7	Koper Bay, Slovenia
Gelidium crinale	Battelli10	Koper Bay, Slovenia
Gelidium crinale	BACI6313	Cala Incina, Italy
Gelidium crinale	BACO1194	Cozze loc., Calarena, Bari, Italy
Gelidium crinale	BACO874	Cozze loc., Calarena, Bari, Italy
Gelidium crinale	BACO1195	Cozze loc., Calarena, Bari, Italy
Gelidium crinale	BALF12101	Lido S. Francesco, Bari, Italy
Gelidium crinale	BALF12102	Lido S. Francesco, Bari, Italy
Gelidium crinale	FGVA23104	Lago de Varano loc., Contrada Chianca, Foggia, Italy
Gelidium crinale	FGVA23105	Lago de Varano loc., Contrada Chianca, Foggia, Italy
Gelidium crinale	FGVA23101	Lago de Varano loc., Crocifisso, Foggia, Italy
Gelidium crinale	FGVA23102	Lago di Varano loc., Crocifisso, Foggia, Italy
Gelidium crinale	FGVA23103	Lago di Varano loc., Crocifisso, Foggia, Italy
Gelidium crinale	BACP25213	Punta della Contessa, Italy
Gelidium crinale	ATVA 01 R1	Varano, Ancona, Italy
Gelidium crinale	TABAT1012	Battendieri, Taranto, Italy
Gelidium crinale	TACI10121	Cimino, Taranto, Italy
Gelidium crinale	TACI6102	Mar Piccolo loc. Cimino, Taranto, Italy
Gelidium crinale	TACI6101	Mar Piccolo loc., Cimino, Taranto, Italy
Gelidium crinale	-	Awaji Island, Hyogo Prefecture, Japan
Gelidium crinale	CNU063322	Awase, Okinawa, Japan
Gelidium crinale	CNU009062	Dugok, Namhaegun, Korea
Gelidium crinale	CNU009063	Dugok, Namhaegun, Korea
Gelidium crinale	CNU009065	Dugok, Namhaegun, Korea
Gelidium crinale	CNU000123	Jeoncheon, Donghae, Korea
Gelidium crinale	210233	Jumunjin, Gangneung, Korea
Gelidium crinale	G2656	Muchangpo, Boryeong, Korea
Gelidium crinale	CNU068531	Tonggumi, Ulleungdo, Korea
Gelidium crinale	CNU068531-1	Tonggumi, Ulleungdo, Korea
Gelidium crinale	CNU058980	Myeongsasipri, Wando, Korea
Gelidium crinale	CNU058992	Myeongsasipri, Wando, Korea
Gelidium crinale	CNU063472-1	Myeongsasipri, Wando, Korea
Gelidium crinale	CNU081484	Geumgap, Jindo, Korea
Gelidium crinale	CNU081485	Geumgap, Jindo, Korea
Gelidium crinale	G6131	Bumseom, Jeju, Korea
Gelidium crinale	G6135	Bumseom, Jeju, Korea
Gelidium crinale	G3923	Pyeongdae, Jeju, Korea
Gelidium crinale	ASB123	Northland, North Island, New Zealand
Gelidium crinale	ASD265	Tapeka Point, North Island, New Zealand
Gelidium crinale	PR	Playa Esperanza, Manati, Puerto Rico
Gelidium crinale	CNU031021	Sentosa Island, Singapore
Gelidium crinale	SANT-Algae 28095	A Toxa, Pontevedra, Spain
Gelidium crinale	PC0165830 (LLG5929)	Agiti Bidea, Euskadi, Basque Country, Spain
Gelidium crinale	-	Aramar, Asturias, Spain
Gelidium crinale	-	Aramar, Asturias, Spain

Gelidium crinale	-	La Arana, Malaga, Spain
Gelidium crinale	SANT-Algae 25618	La Franca, Asturias, Spain
Gelidium crinale	-	La Garita, Canary Island, Spain
Gelidium crinale	SANT-Algae 24719	Perbes, A Corunha, Spain
Gelidium crinale	SANT-Algae 24631	Playa de Langre, Cantabria, Spain
Gelidium crinale	SANT-Algae 26556	Punta Plata, Cadiz, Spain
Gelidium crinale	G6359	Tarifa, Cadiz, Spain
Gelidium crinale	SANT-Algae 25147	Zumaia, Guipuzcoa, Spain
Gelidium crinale	ODC605	Kunduchi, Tanzania
Gelidium crinale	-	Ilfracombe, Devon, UK
Gelidium crinale	G6292	Sidmouth, Devon, UK
Gelidium crinale	biNC	Beaufort Inlet, North Carolina, USA
Gelidium crinale	bsNC	Bogue Sound, North Carolina, USA
Gelidium crinale	ffNC	Fort Fisher, North Carolina, USA
Gelidium crinale	miNC	Masonboro Inlet, North Carolina, USA
Gelidium crinale	riNC	Radio Island, North Carolina, USA
Gelidium crinale	GamerNC2	Stump Sound, North Carolina, USA
Gelidium crinale	-	Port Aransas, Texas, USA
Gelidium crinale	LB2321	Port Aransas, Texas, USA
Gelidium crinale	-	Marineland, Florida, USA
Gelidium crinale	CNU080561	Nam O, Da Nang, Vietnam
Gelidium crinale	CNU080561-2	Nam O, Da Nang, Vietnam
Gelidium crinale	CNU080562	Nam O, Da Nang, Vietnam
Gelidium crinale	CNU081040	Nam O, Da Nang, Vietnam
Gelidium crinale	CNU081039	Nam O, Da Nang, Vietnam
Gelidium crinale	CNU081039-1	Nam O, Da Nang, Vietnam
Pterocladia heteroplatos	PC0452740	Malabar Hill, Bombay, India (cotype of Gelidium heteroplatos)
Pterocladia heteroplatos	LNR-50330	Malabar Hill, Bombay, India (type Gelidium heteroplatos; 5275)

Dash (-) indicates not applicable.

Collection date	Latitude	Longitude	COI-5P haplotype	COI-5P	rbcL
2013.06.26	-2.803566	-41.729772	-	KX574741	KX555615
2013.05.23	-12.818986	-38.218708	-	KX574739	KX555613
2014.01.23	-33.914876	151.261489	C28	**new**	-
-	-31.51435	159.043857	C28	HQ412466	-
-	-35.171169	150.69129	C29	HQ412467	AY350781
-	-32.023915	115.451143	-	-	AF308788
-	-32.023915	115.451143	-	-	AY350780
2002.08.14	-32.01665	115.499184	C30	HQ412464	HQ412492
2002.08.14	-32.01665	115.499184	C30	HQ412465	HQ412493
2009.05.08	-23.369789	-44.797486	C11	KT207975	-
2009.05.08	-23.369789	-44.797486	C11	KT207977	-
2009.05.08	-23.369789	-44.797486	C10	KT207986	-
2009.04.09	-23.500559	-45.120686	C10	KT207985	-
2009.04.09	-23.500559	-45.120686	C10	KT207988	KT208100
2009.09.04	-23.496885	-45.146814	C10	KT207984	-
2010.06.30	-25.156378	-47.916849	C11	KT207991	-
2009.10.20	-24.22219	-46.851173	C10	KT207992	-
2010.05.14	-24.22219	-46.851173	C10	KT207976	-
2009.10.20	-24.195884	-46.801091	C12	KT207980	-
2009.11.01	-23.986814	-46.186454	C11	KT207982	KT208099
2009.11.01	-23.986814	-46.186454	C11	KT207983	-
2012.05.09	-20.729907	-40.52384	C11	KT207979	-
2010.10.05	-20.189166	-40.190153	C13	KT207989	-
2012.05.05	-20.806266	-40.607948	C10	KT207981	-
2012.05.05	-20.806266	-40.607948	C10	KT207987	-
2014.07.12	-25.573319	-48.30982	C10	KX574726	KX555610
2014.05.12	-16.727269	-39.12293	C11	KX574725	-
2014.01.03	-10.938186	-37.018139	C11	KX574724	-
2013.04.24	-3.716765	-38.511207	C11	KX574722	KX555609
2011.10.24	-22.732934	-41.957132	C11	KT207990	-
2014.03.30	-5.875388	-35.174312	C11	KX574723	-
2009.08.20	-5.268898	-35.382644	C11	KT207978	-
2014.11.17	-27.149052	-109.432405	C31	**new**	-
2010.10.11	36.07442	120.447879	C24	JX096528	JX096509
2010.10.11	36.07442	120.447879	-	-	JX096510
2009.05.11	19.190894	109.679307	C26	JX096529	JX096511
2009.05.11	19.190894	109.679307	C26	JX096530	JX096512
2011.09.12	37.531404	121.43423	C24	JX096531	JX096513
2014.12.09	29.987054	122.387513	C24	**new**	-
2014.12.09	29.987054	122.387513	C24	**new**	-
2009.08.12	48.44114333	-4.788093333	C1	**new**	-
2019.07.13	42.5294	3.08459	C1	**new**	-
2019.07.13	42.5294	3.08459	C1	**new**	-
2019.07.13	42.5294	3.08459	C1	**new**	-
2019.07.13	42.5294	3.08459	C1	**new**	-
2019.04.17	48.728624	-3.987586	C1	**new**	-
2019.04.17	48.728624	-3.987586	C1	**new**	-
2019.04.17	48.728624	-3.987586	C1	**new**	-
2019.04.17	48.728624	-3.987586	C1	**new**	-
2019.04.17	48.728624	-3.987586	C1	**new**	-
2019.04.17	48.728624	-3.987586	C1	**new**	-

2019.04.17	48.728624	-3.987586	C1	**new**	-
2019.04.19	48.727303	-4.010466	C1	**new**	-
2019.04.19	48.727303	-4.010466	C1	**new**	-
2019.04.19	48.727303	-4.010466	C1	**new**	-
2008.04.23	22.232961	114.252579	C23	JX096532	-
2008.04.23	22.232961	114.252579	C23	JX096533	JX096514
2008.04.23	22.232961	114.252579	C23	JX096534	JX096515
-	45.544492	13.719717	C15	**new**	-
-	45.544492	13.719717	C4	**new**	-
-	45.544492	13.719717	C1	**new**	-
-	40.987453	17.25807	C4	**new**	-
2011.09.11	41.040142	17.137278	C16	**new**	-
2012.07.08	41.040142	17.137278	C15	**new**	-
2011.09.11	41.040142	17.137278	C17	KU958465	-
2011.10.12	41.137126	16.833893	C17	KU958466	-
2011.10.12	41.137126	16.833893	C17	**new**	-
2011.10.23	41.921243	15.74936	C17	**new**	-
2011.10.23	41.921243	15.74936	C17	**new**	**new**
2011.10.23	41.921243	15.74936	C15	KU958467	KU958473
2011.10.23	41.921243	15.74936	C15	**new**	-
2011.10.23	41.921243	15.74936	C17	**new**	-
-	40.613191	18.026267	C15	**new**	-
-	43.608817	13.480131	C15	**new**	-
2012.10.04	40.494549	17.324101	C15	**new**	-
2012.10.04	40.470094	17.298926	C18	**new**	-
2011.10.06	40.484638	17.282861	C18	**new**	-
2011.10.06	40.484638	17.282861	C18	KU958468	KU958474
1996.10.01	34.436043	134.918617	-	-	AB017679
2014.09.20	26.314546	127.836874	C25	**new**	-
2010	34.768067	127.907885	C22	JX096536	-
2010	34.768067	127.907885	C22	JX096537	-
2010	34.768067	127.907885	C22	JX096538	-
2010.11.25	37.481933	129.158086	C21	JX096539	JX096518
-	37.89443	128.834369	C19	**new**	-
2007.07.29	36.240731	126.529042	C19	HM629863	HM629823
2015.08.06	37.466227	130.835651	C19	**new**	-
2015.08.06	37.466227	130.835651	C19	**new**	-
2014.07.13	34.323063	126.83297	C20	**new**	-
2014.07.13	34.323063	126.83297	C20	**new**	-
2014.10.24	34.323063	126.83297	C20	**new**	-
2020.08.29	34.394401	126.277906	C19	**new**	
2020.08.29	34.394401	126.277906	C19	**new**	
2009.07.05	33.216005	126.518644	C19	JX096535	JX096517
2009.08.23	33.216005	126.518644	-	-	JX096516
2010.05.29	33.534076	126.841135	C19	JX096540	-
2001.05.07	-35.2533	174.133	-	-	AY648021
2003.10.29	-35.245053	174.118415	C28	KF381378	KF381387
-	18.484897	-66.525684	C14	HQ412463	U00983
2012.10.14	1.247296	103.823797	C27	**new**	-
2013.04.24	42.487686	-8.845649	C1	KJ179937	-
2018.06.24	43.309254	-2.071842	C1	**new**	-
-	43.611892	-5.782661	-	-	AF308791
-	43.611892	-5.782661	-	-	AF308792

-	36.712244	-4.321741	-	-	AF308789
2011.03.23	43.394159	-4.571691	C1	KJ179935	-
-	28.007096	-15.376164	-	-	AF308793
2011.01.20	43.375864	-8.215395	C1	KJ179932	-
2010.11.06	43.477	-3.694	C1	KJ168044	-
2011.02.19	36.531407	-6.306373	C3	KJ179933	-
2010.04.12	36.009548	-5.609565	C3	JX096541	JX096519
2011.03.18	43.299714	-2.261365	C2	KJ179934	-
1997.07.10	-6.658452	39.218958	C32	**new**	**new**
-	51.209646	-4129078	-	-	AF308786
2010.04.04	50.677281	-3.237448	C1	JX096542	JX096520
-	34.694337	-76.667863	C7	HQ412458	AF308795
1991.02.16	34.708425	-76.858863	C9	HQ412460	HQ412489
1991.05.14	33.970834	-77.916451	C6	HQ412461	HQ412490
-	34.180209	-77.803362	C6	HQ412457	U00981
1991.02.16	34.713631	-76.680296	C6	HQ412459	HQ412488
2003.07.11	34.482858	-77.435761	C8	HQ412462	HQ412491
-	27.83319	-97.03972	-	-	U00982
-	27.83319	-97.03972	C5	JX096543	JX096521
-	29.669075	-81.212201	-	-	AF308794
2018.05.17	16.119194	108.130703	C25	**new**	-
2018.05.17	16.119194	108.130703	C25	**new**	-
2018.05.17	16.119194	108.130703	C25	**new**	-
2019.02.25	16.119194	108.130703	C25	**new**	**new**
2019.02.25	16.119194	108.130703	C25	**new**	-
2019.02.25	16.119194	108.130703	C25	**new**	-
1927	18.940958	72.795203	C33	**new**	**new**
1927	18.940958	72.795203	-	-	**new**
